# Degradable Spirocyclic Polyacetal-Based Core-Amphiphilic Assemblies for Encapsulation and Release of Hydrophobic Cargo

**DOI:** 10.3390/nano11010161

**Published:** 2021-01-10

**Authors:** Brandon Andrade-Gagnon, Marilyne Bélanger-Bouliga, Phuong Trang Nguyen, Thi Hong Diep Nguyen, Steve Bourgault, Ali Nazemi

**Affiliations:** Department of Chemistry, Université du Québec à Montréal, C.P.8888, Succursale Centre-Ville, Montréal, QC H3C 3P8, Canada; andrade-gagnon.brandon@courrier.uqam.ca (B.A.-G.); belanger-bouliga.marilyne@courrier.uqam.ca (M.B.-B.); phuong.t.ngn@gmail.com (P.T.N.); nguyen.thi_hong_diep@courrier.uqam.ca (T.H.D.N.); bourgault.steve@uqam.ca (S.B.)

**Keywords:** pH-degradable nanoparticles, spirocyclic polyacetals, drug delivery, cytocompatibility, stimuli-responsive nanomaterials

## Abstract

Polymeric nanomaterials that degrade in acidic environments have gained considerable attention in nanomedicine for intracellular drug delivery and cancer therapy. Among various acid-degradable linkages, spirocyclic acetals have rarely been used to fabricate such vehicles. In addition to acid sensitivity, they benefit from conformational rigidity that is otherwise not attainable by their non-spirocyclic analogs. Herein, amphiphilic spirocyclic polyacetals are synthesized by Cu-catalyzed alkyne–azide “click” polymerization. Unlike conventional block copolymers, which often form core–shell structures, these polymers self-assemble to form core amphiphilic assemblies capable of encapsulating Nile red as a hydrophobic model drug. In vitro experiments show that while release from these materials can occur at neutral pH with preservation of their integrity, acidic pH accelerates efficient cargo release and leads to the complete degradation of assemblies. Moreover, cellular assays reveal that these materials are fully cytocompatible, interact with the plasma membrane, and can be internalized by cells, rendering them as potential candidates for cancer therapy and/or drug delivery.

## 1. Introduction

Drug delivery systems are a necessity nowadays due to the limitations of most drugs, which often include low water solubility, high toxicity, low metabolic stability, and poor bioavailability. Drug delivery systems can act by one of these mechanisms: (i) increasing the hydrophilicity of drugs, (ii) limiting drug degradation and elimination, and (iii) enhancing their bioavailability and accumulation at the target site [1,2]. In the past few decades, the emergence of controlled polymerization techniques has enabled the synthesis of polymers of various chemical structures, topologies, and functions [3,4,5,6,7]. This, coupled with the ability to self-assemble amphiphilic polymers to nano-sized materials with a variety of morphologies, including spherical micelles and vesicles, one-dimensional worms and cylinders as well as toroids, has rendered polymer-based nanomaterials promising candidates for applications in nanomedicine [8,9]. This includes their use as carriers for hydrophilic and hydrophobic small molecules, proteins, polynucleotides, and imaging contrast agents [10,11]. In this context, degradable polymeric nanomaterials, in which the release of the encapsulated cargo can be triggered in response to an external stimulus, are of significant interest. To date, materials responsive to almost every conceivable stimulus, including temperature, light, mechanical force, pH, and electron transfer (oxidation−reduction) have been developed [12,13,14]. Among these triggers, a change in the acidity, i.e., pH, is particularly interesting for developing responsive/degradable nanomaterials. This is because, when it comes to the delivery of any drug, the pH change from the acidity in the stomach (pH = 2) to the intestine (pH = 5–8) has to be considered [15]. In addition, it is known that the extracellular pH of tumor tissues is often 0.5–1.0 units lower than that of the blood and normal tissues [16]. Moreover, upon cell uptake via endocytosis [17], the pH drops from neutral to 5.5–6.0 in endosomes and approaches to a value of 4.5–5.0 in late endosomes and lysosomes [17]. Therefore, polymeric materials that could degrade and release their cargo in an acidic environment are attractive candidates for the delivery of therapeutics intracellularly and to cancerous cells.

pH-responsive materials are generally divided into two categories. The first class are those containing ionizable functional groups, such as amines and carboxylic acids, which often undergo conformational changes upon (de)protonation without any covalent bond cleavage [13]. The second group is characterized by the presence of linkages, such as acetal, imine, ketal, hydrazine, *β*-thio ester, and *cis*-aconityl, that undergo covalent bond cleavage due to pH variations [18]. Among these pH-degradable linkages, acetals have attracted significant attention because their cleavage often results in the formation of charge-neutral and potentially nontoxic products [19,20]. In fact, a variety of linear and branched polymers as well as dendritic structures with acetal functional groups have been developed. In these materials, the incorporation of acetals within the polymer backbone [21,22,23], in their side chain as pendant groups [24,25,26] or as branching units [27,28,29], has been employed to access scaffolds with tunable degradability.

While it is possible to synthesize both acyclic and cyclic acetals, spirocyclic conformation confers rigidity to the polymer backbone as a result of its restricted conformational flexibility and, hence, results in enhanced polymer thermal stability [30,31,32]. In this context, spirocyclic polyacetals with various chemical structures have been synthesized via different polymerization methods. This includes direct polyacetalization [33,34,35,36,37] and polytransacetalization [38] of carbonyl/acetal and polyol monomers, respectively, as well as polymerization of pre-synthesized spirocyclic acetal monomers via nucleophilic substitution [39] and polycondensation reactions [32,40,41,42]. In these examples, the focus has been principally on fine-tuning the thermal and rheological properties of the resulting polymers and their potential use as replacements for existing thermoplastic and thermoset commodity polymers. Thus far, hydrophobic and rigid spirocyclic polyacetals with limited solubilities in organic and aqueous solvents have been obtained. This has posed a significant hurdle in the application of spirocyclic polyacetals in nanomedicine.

**Scheme 1:**
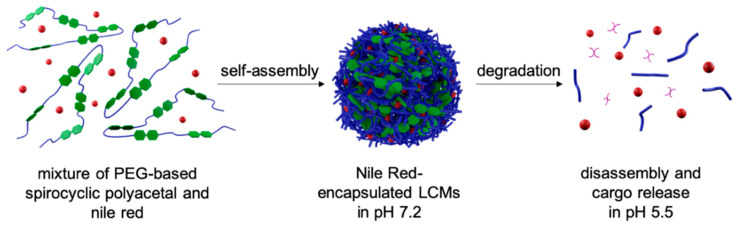
A schematic illustration of the Nile red-loaded core amphiphilic assembly formation using poly(ethylene glycol) (PEG)-based spirocyclic polyacetals and their pH-triggered disassembly and cargo release.

Herein, we report the efficient synthesis of poly(ethylene glycol) (PEG)-based spirocyclic polyacetals using copper(I)-catalyzed alkyne–azide cycloaddition (CuAAC) “click” polymerization as an alternative method to the existing approaches to access this class of polymers. Owing to the rigidity and hydrophobicity of the spirocyclic linkages, we demonstrate that these polymers undergo self-assembly to form core-amphiphilic spherical large-compound micelles (LCM) [8] capable of encapsulating the hydrophobic dye Nile red, used as a model drug. These micelles degrade effectively in an acidic environment (pH = 5.5) to release their encapsulated cargo (Scheme 1). In addition, these LCMs are fully cytocompatible, interact avidly with the plasma membrane, and can be uptaken by cells. To our knowledge, this is the first example of an amphiphilic spirocyclic polyacetal capable of forming self-assembled materials with potential application as a drug delivery vehicle.

## 2. Materials and Methods 

All chemicals were purchased from Sigma-Aldrich (St. Louis, MO, USA) or Alfa Aesar (Haverhill, MA, USA) and were used without further purification unless otherwise noted. Anhydrous *N,N*-dimethylformamide (DMF) was obtained from a solvent purification system using aluminum oxide columns. The reactions were mostly performed in air without the use of air- and moisture-free techniques using reagent grade solvents. The polymerization reactions were performed under argon using Schlenk line techniques and anhydrous DMF. Dialyses were performed using Spectra/Por regenerated cellulose membranes with a 3500 g/mol molecular weight cut-off (MWCO). ^1^H and ^13^C NMR spectra were recorded at 300 and 75 MHz, respectively, using CDCl_3_ as solvent. Chemical shifts are reported in delta (δ) units, expressed in parts per million (ppm). Coupling constants (*J*) are expressed in hertz (Hz). The number-average molecular weight (*M*_n_), weight-average molecular weight (*M*_w_), and dispersity indices (*Đ*) of polymers were determined by size exclusion chromatography (SEC) using an EcoSEC HLC-8320 (Tosoh Bioscience, Tokyo, Japan) instrument equiped with two TSKgel Alpha-M, 13 µm columns (7.8 mm ID × 30 cm L) and a TSKgel Alpha Guardcolumn (6.0 mm ID × 4 cm L) calibrated with poly(methyl methacrylate) standards in DMF containing 10 mM LiBr at 60 °C. The samples were prepared at a concentration of 2 mg/mL in DMF and filtered through a 0.22 μm PTFE syringe filter prior to injection. The data were acquired at a flow rate of 1 mL/min at 60 °C. Dynamic light scattering (DLS) data were obtained using a Zetasizer Nano ZS instrument from Malvern Instruments (Malvern, UK) at a polymer concentration of 0.05 mg/mL. Fluorescence spectroscopy was performed with a Perkin Elmer LS45 Spectrofluorometer (Waltham, MA, USA). Samples were analyzed at a polymer concentration of 0.05 mg/mL. The excitation wavelength for acquiring the emission spectra was set at 552 nm. Differential scanning calorimetry (DSC) data were obtained using a DSC1 Mettler Toledo instrument (Columbus, OH, USA) with a heating/cooling rate of 10 °C/min between −30 and 100 °C under nitrogen atmosphere. The midpoint glass transition temperature (*T*_g_) values were extracted from the second heating cycle. Atomic force microscopy (AFM) data were obtained using a Veeco/Bruker Multimode AFM (Billerica, MA, USA). One drop of the sample (0.05 mg/mL) was deposited on a clean mica surface (12 mm diameter) and was left to dry overnight. They were then used for imaging the next day. Lastly, the samples for the transmission electron microscopy (TEM) were prepared by drop casting one drop (ca. 20 μL) of the colloidal particle solution (1 mg/mL) onto a formvar carbon-coated copper grid rested on a piece of filter paper, which were left to dry overnight. The measurements were performed on a Joel JEM-2100F instrument (Tokyo, Japan) at 80 kV. High-resolution mass spectrometry (HRMS) was performed using a Liquid Chromatography Mass Spectrometry Time of Flight (LC-MS TOF) mass analyzer (Agilent Technologies Santa Clara, CA, USA) in the electrospray mode.

### 2.1. Synthesis of Compound ***2***

Compound **1** [43] (1.6 g, 4.5 mmol, 1.0 equiv.) was dissolved in acetonitrile (60 mL) in a 100 mL round bottom flask. Potassium carbonate (6.2 g, 45 mmol, 10 equiv.) was added slowly to the stirring solution over 10 min. Propargyl bromide (5.4 g, 45 mmol, 10 equiv.) was then added slowly and the solution was stirred under reflux (86 °C) for 20 h. After the removal of the round bottom flask from the oil bath, the solution was cooled down, after which acetonitrile was removed on a rotary evaporator. The residue was dissolved in equal in water/dichloromethane and transferred to a separatory funnel. The aqueous phase was extracted with dichloromethane (3 × 30 mL). The combined organic layers were washed with brine (1 × 30 mL), dried over magnesium sulfate, filtered, and concentrated. The product was a light-yellow solid (1.2 g, yield = 64%). ^1^H NMR (300 MHz, CDCl_3_, δ): 7.46 (d, *J* = 9 Hz, 2H, Ar**H**), 7.00 (d, *J* = 9 Hz, 2H, Ar**H**), 5.44 (s, 2H, C**H**–O_2_), 4.86 (d, 2H, *J* = 9 Hz, O–C**H_2_**–C), 4.70 (d, *J* = 3 Hz, 4H, C**H_2_**C≡CH), 3.87–3.80 (m, 4H, O–C**H_2_**–C), 3.64 (m, 2H, O–C**H_2_**–C), 2.54 (t, 3H, *J* = 3 Hz, C**H_2_**C≡C**H**). ^13^C NMR (75 MHz, CDCl_3_, δ): 158.1, 131.4, 127.4, 114.7, 102.0, 78.4, 75.7, 71.1, 70.6, 55.8, and 32.4. HRMS (EI, *m*/*z*): [M + H]^+.^ Calculated for, C_25_H_25_O_6_ 421.1651; found, 421.1643.

### 2.2. Synthesis of Compound ***3***

This compound was synthesized according to a previously published procedure [44]. Polyethylene glycol (*M*_n_ = 400 g/mol, 5.0 g, 12 mmol, 1.0 equiv.) was dissolved in dichloromethane (100 mL). Triethylamine (5.0 g, 50 mmol, 4.0 equiv.) was added slowly to the stirring solution, followed by 4-dimethylaminopyridine (3.0 g, 25 mmol, 2.0 equiv.) and *p*-toluenesulfonyl chloride (7.1 g, 38 mmol, 3.0 equiv.). The reaction was stirred at room temperature overnight. The organic layer was washed with HCl (2 M, 2 × 30 mL), water (1 × 30 mL), brine (1 × 30 mL), dried over magnesium sulfate, filtered and concentrated to remove dichloromethane. The product was a dark orange viscous oil (7.4 g, yield = 83%). ^1^H NMR (300 MHz, CDCl_3_, δ): 7.77 (d, *J* = 6 Hz, 2H, Ar**H**), 7.33 (d, *J* = 6 Hz, 2H, Ar**H**), 4.14 ppm (t, *J* = 6 Hz, 4H, C**H_2_**–OTs), 3.76–3.56 (m, 36H, O–C**H_2_**–C**H_2_**), 2.43 (s, 6H, CH_3_).

### 2.3. Synthesis of Compound ***4***

This compound was synthesized according to a previously published procedure [44]. Polyethylene glycol (*M*_n_ = 600, 5.0 g, 8.3 mmol, 1.0 equiv.) was dissolved in a 250 mL round bottom flask in dichloromethane (100 mL). Triethylamine (3.4 g, 33 mmol, 4.0 equiv.) was added slowly to the stirring solution, followed by 4-dimethylaminopyridine (2.0 g, 17 mmol, 2.0 equiv.) and *p*-toluenesulfonyl chloride (4.8 g, 25 mmol, 3.0 equiv.). The reaction was stirred at room temperature overnight. The organic layer was washed with HCl (2M, 2 × 30 mL), water (1 × 30 mL), brine (1 × 30 mL), dried over magnesium sulfate, filtered and concentrated to remove dichloromethane. The product was a dark orange viscous oil (4.1 g, yield = 54%). ^1^H NMR (300 MHz, CDCl_3_, δ): 7.75 (d, *J* = 6, 2H Hz, Ar**H**), 7.31 (d, *J* = 6 Hz, 2H, Ar**H**), 4.12 (t, *J* = 6 Hz, 4H, C**H_2_**-OTs), 3.66–3.54 (m, 56H, O–C**H_2_**–C**H_2_**), 2.41 (s, 6H, CH_3_).

### 2.4. Synthesis of Compound ***5***

Compound **3** (7.4 g, 10 mmol, 1.0 equiv.) was dissolved in a 100 mL round bottom flask in DMF (70 mL). Sodium azide (14 g, 2.1 × 10^2^ mmol, 20 equiv.) was added slowly to the stirring solution. The reaction mixture was stirred at 80 °C overnight. After the solution was cooled down, DMF was removed using a rotary evaporator. Portions of water/dichloromethane were added to the round bottom flask and transferred to a separatory funnel. Aqueous layer was extracted with dichloromethane (3 × 30 mL). The organic layers were combined and washed with brine (1 × 30 mL), dried over magnesium sulfate, filtered, and concentrated to remove dichloromethane. Product was obtained as a light orange viscous oil (3.2 g, 68% yield). ^1^H NMR (300 MHz, CDCl_3_, δ): 3.80–3.54 (m, 32H, O–C**H**_2_–C**H**_2_), 3.27 ppm (t, *J* = 6 Hz, 4H, C**H_2_**–N_3_). ^13^C NMR (75 MHz, CDCl_3_, δ): 70.7, 70.7, 70.6, 70.6, 70.0, 50.7 ppm.

### 2.5. Synthesis of Compound ***6***

Compound **4** (4.1 g, 4.5 mmol, 1.0 equiv.) was dissolved in a 100 mL round bottom flask in DMF (60 mL). Sodium azide (5.8 g, 90 mmol, 20 equiv.) was added slowly to the stirring solution. The reaction mixture was stirred at 80 °C overnight. After the solution was cooled down, the DMF was removed using a rotary evaporator. Equal portions of water/dichloromethane were added to the round bottom flask and transferred to a separatory funnel. The aqueous layer was extracted with dichloromethane (3 × 30 mL). The organic layers were combined and washed with brine (1 × 30 mL), dried over magnesium sulfate, filtered, and concentrated to remove dichloromethane. The product was obtained as a light orange viscous solid (2.0 g, yield = 67%). ^1^H NMR (300 MHz, CDCl_3_, δ): 3.84–3.57 (m, 52H, O–C**H_2_**–C**H_2_**), 3.31 ppm (t, *J* = 6 Hz, 4H, C**H_2_**–N_3_). ^13^C NMR (75 MHz, CDCl_3_, δ): 70.7, 70.7, 70.6, 70.6, 70.0, 50.7 ppm.

### 2.6. Synthesis of Polymer ***7***

A dry Schlenk flask was first purged with argon. Compound **5** (0.24 g, 0.53 mmol, 1.0 equiv.) was then added directly to the Schlenk flask, followed by compound **2** (0.24 g, 0.56 mmol, 1.05 equiv.), and anhydrous DMF (5 mL). While the mixture was stirring under argon, *N,N,N’,N’*-tetramethylethylenediamine (TMEDA) (16 mg, 0.13 mmol, 0.25 equiv.) was added. The solution was saturated with argon. Copper(I) bromide (19 mg, 0.13 mmol, 0.25 equiv.) was added and the solution was freeze-pump-thawed three times to remove oxygen. The reaction mixture was placed in an oil bath pre-heated at 60 °C and stirred for 24 h. After the solution was cooled down, it was dialyzed against DMF for 24 h using a 3.5 K MWCO membrane where the dialysate was changed every 6 h. Afterwards, the DMF was removed under reduced pressure and the product was dissolved in chloroform. The solution was placed in an Erlenmeyer flask, where ammonia was added and stirred for 10 min (to remove the residual Cu catalyst). Both layers were transferred to a separatory funnel, where the organic layer was removed. The organic layer was repetitively placed in ammonia until the color remained constant. The organic layer was washed with brine (3 × 30 mL) dried over magnesium sulfate, filtered, and concentrated to remove chloroform. Product was obtained as a light-yellow sticky solid (0.26 g, yield = 56%). ^1^H NMR (300 MHz, CDCl_3_, δ): 7.79 (s, 2H, triazole ring) 7.38 (d, *J* = 9 Hz, 2H, Ar**H**), 6.95 (d, *J* = 9 Hz, 2H, Ar**H**), 5.37 (s, 2H, C**H**–O_2_), 5.17 ppm (s, 4H, C–C**H_2_**–O), 4.78 ppm (br. D, 2H, O–C**H_2_**–C), 4.50 (br. S, 4H, O–CH_2_–C**H_2_**–N), 3.83-3.51 (m, 40H, C**H_2_** in PEG backbone and the rest of protons in spirocyclic skeleton). SEC: *M*_n_ = 15,900, *Đ* = 2.47.

### 2.7. Synthesis of Polymer ***8***

A dry Schlenk flask was first purged with argon. Compound **6** (0.31 g, 0.48 mmol, 1.0 equiv.) was then added directly to the Schlenk flask, followed by compound **2** (0.21 g, 0.51 mmol, 1.05 equiv.) and anhydrous DMF (5 mL). While the mixture was stirring under argon, TMEDA (14 mg, 0.12 mmol, 0.25 equiv.) was added. The solution was saturated with argon. Copper(I) bromide (17 mg, 0.12 mmol, 0.25 equiv.) was added and the solution was freeze-pump-thawed three times to remove oxygen. The reaction mixture was placed in an oil bath pre-heated at 60 °C and stirred for 24 h. After the solution was cooled down, it was dialyzed against DMF for 24 h using a 3.5 K MWCO membrane in, where the dialysate was changed every 6 h. Afterwards, DMF was removed under reduced pressure and the product was dissolved in chloroform. The solution was placed in an Erlenmeyer flask, where ammonia was added and stirred for 10 min (to remove the residual Cu catalyst). Both layers were transferred to a separatory funnel, where the organic layer was removed. The organic layer was repetitively placed in ammonia until the color remained constant. The organic layer was washed with brine (3 × 30 mL) dried over magnesium sulfate, filtered, and concentrated to remove chloroform. The product was obtained as a light-yellow sticky solid (0.34 g, yield = 65%). ^1^H NMR (300 MHz, CDCl_3_, δ): 7.81 (s, 2H, triazole ring) 7.37 (d, *J* = 9 Hz, 2H, Ar**H**), 6.95 (d, *J* = 9 Hz, 2H, Ar**H**), 5.37 (s, 2H, C**H**–O_2_), 5.17 ppm (s, 4H, C–C**H_2_**–O), 4.78 ppm (br. D, 2H, O–C**H_2_**–C), 4.50 (br. S, 4H, O–CH_2_–C**H_2_**–N), 3.83–3.51 (m, 58H, C**H_2_** in PEG backbone and the rest of protons in spirocyclic skeleton). SEC: *M*_n_ = 73,000, *Đ* = 3.07.

### 2.8. Procedure for the Self-Assembly of ***7*** and ***8***

To obtain 1 mg/mL solutions of the self-assembled materials, 5 mg of polymers **7** and **8** were separately dissolved in dimethyl sulfoxide (DMSO) (0.5 mL) and sonicated for 5 min to ensure their complete dissolution. To this solution, filtered nanopure water (4.5 mL) was added dropwise while stirring. Following the formation of particles, the samples were dialyzed against deionized (DI) water using a 3500 MWCO membrane for 24 h with multiple changes of the dialysate. The resulting assemblies were then used for characterization by DLS, AFM, and TEM.

### 2.9. Procedure for the Encapsulation of Nile Red by Particles Formed by Polymer ***7***

To encapsulate Nile red in the assemblies formed from polymer **7**, the above-mentioned procedure for the particle formation was employed except that the initial solution of **7** in DMSO contained 1% w/w Nile red.

### 2.10. Procedure for the Degradation Study of Polymers ***7*** and ***8*** by ^1^H NMR

To study the degradation of **7** and **8** by ^1^H NMR, these polymers were separately dissolved in trifluoroacetic acid (TFA)-containing deuterated chloroform (CDCl_3_) (80 mM, 1 mL) and NMR spectra were obtained at the specified time points. We would like to note that our attempts to carry out this experiment in mixtures of organic solvents, such as dioxane, acetone, and DMSO, and water were met with very limited success as the polymers readily precipitated in such media.

### 2.11. Procedure for Nile Red Release Study

To study the release of Nile red from particles formed by **7**, as detailed above, the preformed dye-encapsulated assemblies (150 μL, 1 mg/mL) were added to phosphate buffer (2.85 mL, 10 mM) at pH 5.5 and 7.4 in quartz cuvettes preheated at 37 °C in a water bath. Fluorescence spectra were acquired at the specified time points, with an excitation wavelength of 552 nm, and the samples were rapidly placed back in water bath to minimize any significant drop in temperature. At the end of the release studies, the same samples were used to measure the size of particles by DLS.

### 2.12. Procedure for Cell Viability Assays

Chinese hamster ovary cells K1 (CHO K1; from ATCC, Manassas, VA, USA) were maintained in Ham’s F12 medium supplemented with 10% fetal bovine serum (FBS), 2 mM *L*-glutamine and antibiotic antimycotic (10,000 UI/mL penicillin, and 10,000 UI/mL streptomycin). The cells were maintained as a monolayer at 37 °C in a humidified atmosphere of 5% CO_2_ and 95% air and passaged by trypsinization when the cells reached around 80% confluence. For the LIVE/DEAD assay, CHO K1 were seeded in 12-well plates at a density of 60,000 cells/well and incubated overnight at 37 °C. The cells were then treated with the direct addition of particle solutions (diluted in PBS) to reach the final desired concentrations. After 24 h incubation, the medium was removed and LIVE/DEAD reagent solution (4 µM ethidium homodimer-1; 2 µM calcein-AM) in sterile PBS was added in each well. After incubation for 45 min, the samples were analyzed using a fluorescent microscope with a 20× objective lens. At least three images per well were taken and processed using ImageJ software. The percentage of cell viability was calculated as the viable cells (green labelled) over the total number of cells (green + red). For the metabolic assay, CHO K1 cells were seeded in black-wall clear-bottom 96-well plates at a density of 30,000 cells/well and incubated overnight at 37 °C in a 5% CO_2_. The cells were treated by the direct addition of particle solutions (diluted in PBS) to reach the final desired concentrations. After 24 h incubation, cellular viability was measured by the resazurin reduction assay. Cell viability (in %) was calculated from the ratio of the fluorescence of the treated sample to the vehicle-treated control cells (PBS). The data of at least three independent experiments were averaged and expressed as the mean ± S.D. The results were analyzed using the Student’s t-test and statistical difference was established at *p* < 0.01. Statistical analysis was performed using Prism 6.0 software.

### 2.13. Procedure for Evaluation of Cellular Uptake

CHO K1 cells were cultured on coverslips for 24 h at 60,000 cells/well in 8-well cell culture chamber. The cells were washed with PBS and incubated with particles (100 μg/mL) at 37 °C for 4 h. The cells were then washed three times with PBS, fixed with 4% paraformaldehyde (Santa Cruz) and stained with 1 µg/mL DAPI (4′,6-Diamidino-2-phenylindole dihydrochloride) for the nucleus and FAST DiO for the plasma membrane. The cover glasses were mounted and the fluorescence was analyzed with a confocal microscope using a 60× oil immersion lens. The images were analyzed using ImageJ software. For flow cytometry analysis, the cells were seeded in 12-well plates at a density of 60,000 cells/well overnight. The cells were treated by the direct addition of dye-loaded particles (diluted in PBS) to reach a final concentration of 100 μg/mL. After incubation for 4 h, the cells were washed 3 times with PBS buffer and harvested. Cells were suspended in PBS buffer prior to flow cytometry analysis.

## 3. Results and Discussion

### 3.1. Synthesis and Characterization of Spirocyclic Polyacetals

**Scheme 2:**
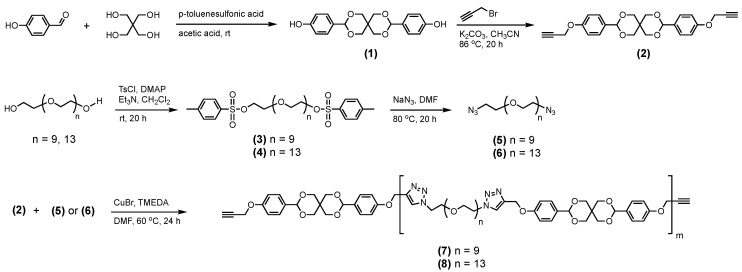
The synthesis of the monomers and the spirocyclic polyacetals. TsCl: *p*-toluenesulfonyl chloride.

To construct our target polymers via CuAAC “click” polymerization, we envisioned the synthesis of dialkyne-decorated spirocyclic acetal and diazide-functionalized PEG monomers. Our synthetic strategy is shown in Scheme 2. The use of starting materials from biorenewable resources is of significant interest as a viable alternative to fossil-based materials due to the ongoing concerns regarding the environmental impact of the latter on the planet. In this context, we chose pentaerythritol and 4-hydroxybenzaldehyde as starting materials for the synthesis of our dialkyne-containing monomer. 4-Hydroxybenzaldehyde is considered as one of the three aromatic 4-hydroxyaldehydes, alongside vanillin and syringaldehyde, derived from lignin, the second most abundant naturally occurring polymer and an excellent source of biobased aromatic feedstocks [45]. Although pentaerythritol is not a bio-sourced compound, it is produced commercially from formaldehyde and acetaldehyde and is an attractive polyol for the synthesis of spirocyclic acetals because of its low cost [34]. Double protection of this compound using 4-hydroxybenzaldehyde under acidic conditions resulted in the formation of spirocyclic acetal (**1**) with phenol functional groups. The subsequent reaction of **1** with propargyl bromide using potassium carbonate as base provided our “clickable” dialkyne-functionalized spirocyclic acetal (**2**) in 64% yield (Scheme 2). To render the final polymers and their corresponding particles dispersible in aqueous media, we chose diazide-functionalized PEG as the complementary monomer to **2**. In order to investigate the effect of hydrophilic segment chain length on the size of the resulting assemblies, PEG with *M*_n_ (number average molecular weight) values of 400 and 600 g/mol were selected, corresponding to the degree of polymerization of 10 and 14, respectively. Ditosylation of these PEG molecules followed by their reaction with sodium azide in DMF afforded the two diazide-decorated PEG target monomers **7** and **8** (Scheme 2).

**Figure 1:**
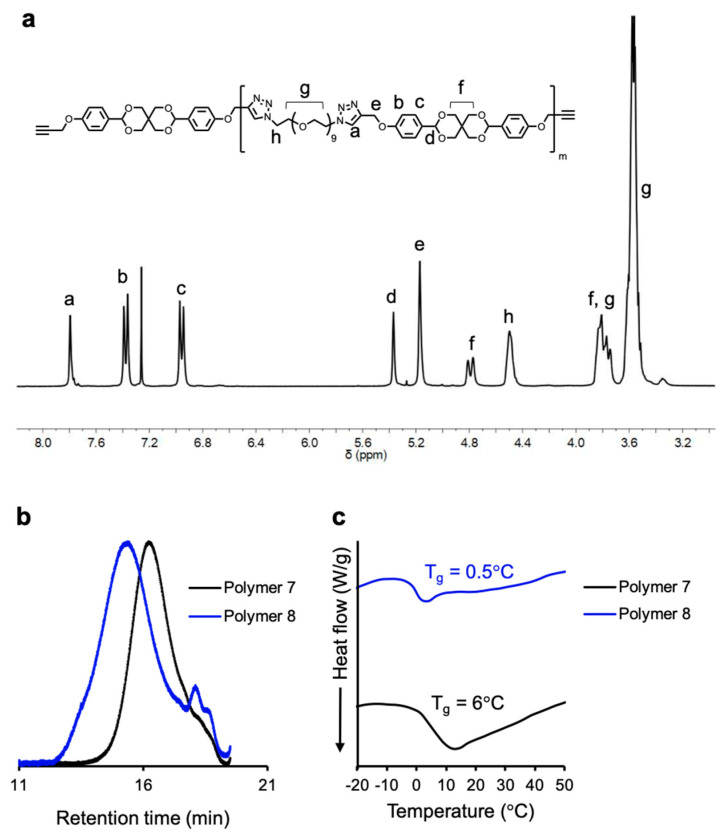
(**a**) ^1^H NMR of **7**; (**b**) refractive index (RI) traces in the SEC analysis of **7** and **8** in DMF; (**c**) DSC analysis of **7** and **8** showing amorphous spirocyclic polyacetals at room temperature.

Having the three monomers in hand, we then synthesized the target spirocyclic polyacetals (**7**) and (**8**) via CuAAC “click” polymerization using CuBr and TMEDA in DMF at 60 °C for 24 h (Scheme 2). In all these reactions, a slight excess of dialkyne-decorated monomer was used to increase the possibility of end-capping the polymers with this functionality and to inhibit the formation of cyclic polymers. However, the formation of the latter cannot be entirely excluded. The resulting polymers were purified by a combination of dialysis against DMF using a 3500 MWCO membrane, to remove any unreacted monomers and low molecular weight oligomers, and washed with ammonia to remove the copper catalyst. The ^1^H NMR analysis of **7** demonstrated the formation of 1,2,3-triazole rings in the polymer backbone as evidenced by the peak at 7.79 ppm (Figure 1a). In addition, the peak at 5.37 ppm, corresponding to the two acetal protons, confirms the presence of the spirocyclic acetals in the polymer structure. Similar ^1^H NMR spectrum was obtained for **8** (Supplementary Materials Figure S10). As shown in Figure 1b, using SEC, *M*_n_ values of 15,900 and 73,000 g/mol with dispersity indices of 2.47 and 3.07 were obtained for **7** and **8**, respectively. It is noteworthy that CuAAC polymerization is a step-growth polymerization for which relatively high *Đ* values are expected. Moreover, the chain length distributions of the PEG starting materials carry on and amplify throughout the polymerization, which contributes significantly to *Đ* broadening of the target polymers. We then used DSC to gain insight into the thermal behaviour of the polymers. The results demonstrate that both **7** and **8** are amorphous with comparable glass transition temperatures (T_g_) at 6 and 0.5 °C, respectively (Figure 1c). As noted earlier, relatively high T_g_ values for this class of polymers are often obtained due to the nature of the spacers and the rigidity of the polymer backbone [34,35,38]. In **7** and **8**, the PEG spacers between the spirocyclic acetal moieties impart adequate flexibility to render them amorphous at room temperature.

**Figure 2:**
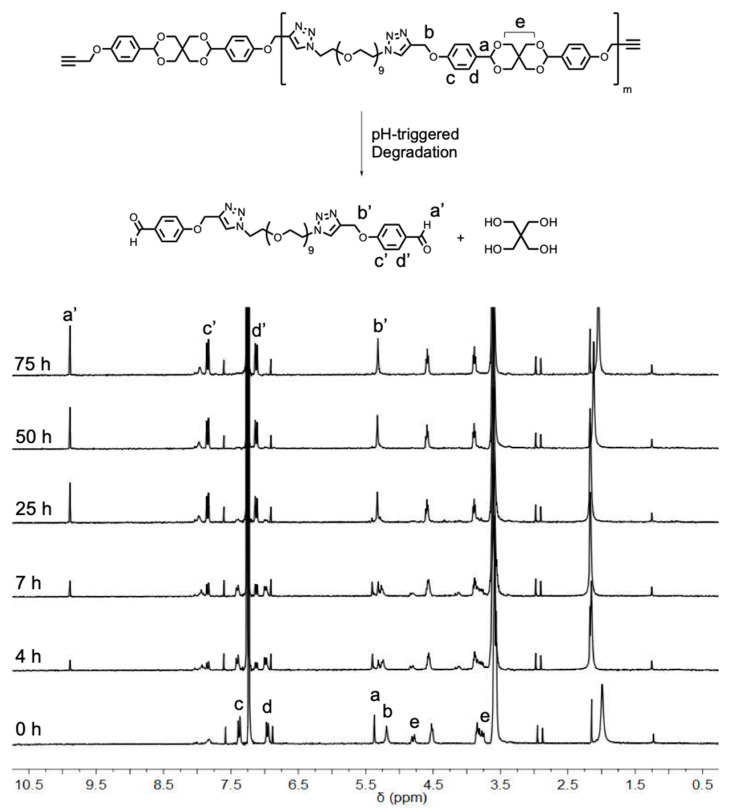
A pH-triggered degradation reaction of **7** in acidic media (**top**) monitored by ^1^H NMR spectroscopy (**bottom**).

Before particle formation and pH-triggered degradation and model drug release experiments, we studied the degradation behaviour of polymers **7** and **8** in acidic organic media. To do this, polymers were dissolved (5 mg/mL) in a CDCl_3_ solution of trifluoroacetic acid (80 mM, 1 mL) and ^1^H NMR spectra were acquired at specified time points. As shown in Figure 2 for **7**, the decrease in intensity of the peak at 5.37 ppm, corresponding to the acetal proton, and the concurrent increase in the intensity of the peak at 9.88 ppm, corresponding to the formation of arylaldehyde derivative, is a strong evidence of the effective breakdown of the polymer backbone. In addition, the initial two sets of doublets at 6.95 and 7.37 ppm, assigned to the aryl groups in the polymer structure, disappear and new doublets appear at 7.11 and 7.83 ppm, which can be attributed to the protons in the resulting arylaldehyde product. Finally, after complete degradation, which takes place in about 75 h, the singlet at 5.17 ppm, corresponding to the CH_2_ between the triazole ring and the spirocyclic moiety, disappears and a new singlet emerges at 5.25 ppm, which is assigned to the same protons now located between the triazole ring and the benzaldehyde derivative in the degradation product. A similar degradation profile was observed for **8** under similar conditions (Figure S11). A peak corresponding to the formed pentaerythritol was not detectable due to its overlap with those of the PEG backbone. However, the degradation of the spirocyclic backbone is evident by the disappearance of the multiplets at 4.80 and 3.80 ppm assigned to its protons. Performing a similar stability experiment for polymer **7** in non-acidic CDCl_3_ revealed no degradation of the polymer backbone during the four-day timeframe of the measurements (Figure S12). We would like to note that our attempts to carry out this experiment in mixtures of organic solvents, such as dioxane, acetone, dimethyl sulfoxide, and water were met with very limited success as the polymers readily precipitated in such media. SEC analysis of these two samples demonstrated the complete degradation of polymer backbones and the formation of low molecular weight oligomers, further confirming the effective breakdown of **7** and **8** under acidic condition (Figure S13).

### 3.2. Self-Assembly of Spirocyclic Polyacetals ***7*** and ***8***

**Figure 3:**
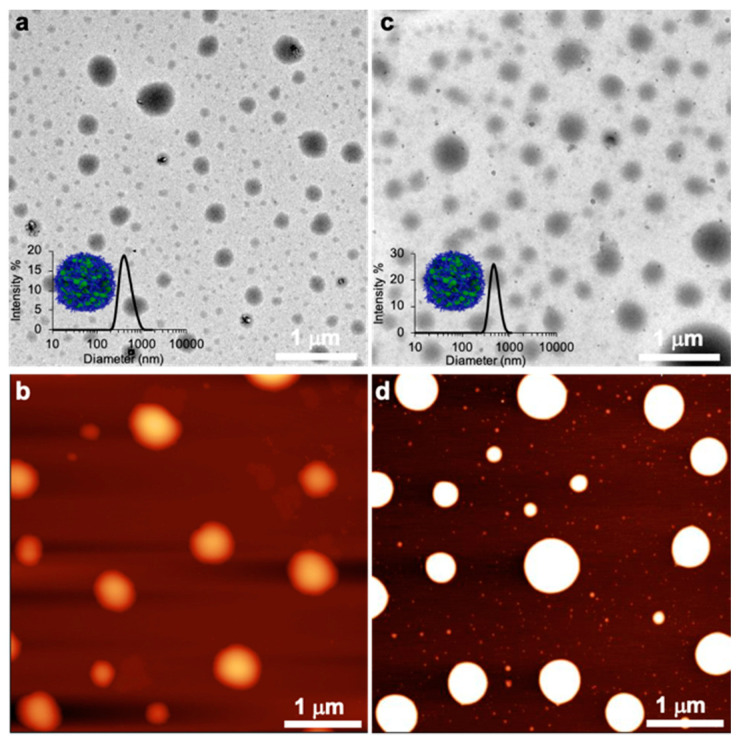
(**a**,**b**) TEM and AFM images of **P1**. Inset showing the DLS data and a cartoon representation of the particles. (**c**,**d**) TEM and AFM images of **P2**. Inset showing the DLS data and a cartoon representation of the particles.

Encouraged by these results, we then investigated the self-assembly behaviour of the polymers. In their structures, the PEG chains serve as the hydrophilic segments. On the other hand, the rigidity of the spirocyclic moieties coupled with the phenyl rings of the 4-hydroxybenzaldehyde protecting groups render them hydrophobic. We envisioned that these hydrophobic nodes on the polymer backbone will be able to drive the phase separation of these polymers in an aqueous environment. As a result of this design, polymers **7** and **8** do not resemble the conventional amphiphilic block copolymers commonly used for the fabrication of soft nanomaterials. To test this hypothesis, polymers (5 mg) were dissolved in DMSO (0.5 mL) and, to these solutions, deionized water (4.5 mL) was added dropwise, with constant stirring. The organic solvent was then removed by dialyzing the solutions against DI water for 24 h with multiple changes of the dialysate. DLS analysis of the samples obtained from **7** and **8** (denoted as **P1** and **P2**, respectively) revealed particles with average hydrodynamic diameters of 400 and 460 nm, respectively (Figure 3a,c, inset). The slightly larger size of **P2** can be potentially attributed to the higher *M*_n_ of **8** compared to that of **7**. Further insight into the morphologies of the structures was obtained by TEM and AFM measurements.

As shown in Figure 3a–d, these analyses demonstrate that both polymers form polydisperse spherical particles whose size is in good agreement with those obtained by DLS. As noted earlier, although **7** and **8** are amphiphilic, they are not comprised of a block structure, but rather they resemble amphiphilic alternating polymers. As a result of this structural feature, **P1** and **P2** cannot adapt the conventional core-corona morphologies with a hydrophobic core and a hydrophilic corona in an aqueous environment. In contrast, it is conceivable that the self-assembled materials formed are similar to large compound micelles (LCMs) with an amphiphilic core, comprised of both the hydrophobic spirocyclic acetals and hydrophilic PEG units (Figure 3a,c, inset). We suggest that it is the association of the hydrophobic spirocyclic nodes on the polymer backbone that act as stitches to form core-amphiphilic LCMs.

### 3.3. Encapsulation and pH-Triggered Release of Nile Red from Particles

**Figure 4:**
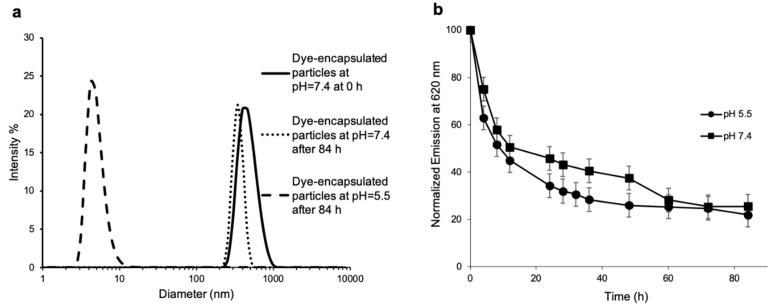
(**a**) DLS data for the Nile red-encapsulated **P1** in water at room temperature as well as those incubated at 37 °C in phosphate buffer at pH 7.4 and 5.5 after 84 h. (**b**) The release profile of Nile red from **P1** at pH 5.5 and 7.4 at 37 °C.

Given the similar morphologies and comparable sizes of **P1** and **P2**, we used **P1** for the rest of the studies to investigate their potential applications as drug delivery vehicles. To do this, we chose Nile red as a model drug that could be encapsulated in the core of particles. It is noteworthy that Nile red is hydrophobic and the amphiphilic core of the micelles, particularly in interactions with the hydrophobic spirocyclic acetal moieties, offer a suitable environment for its encapsulation and minimizes its contact with water. In addition, Nile red is strongly fluorescent when solubilized in a hydrophobic environment such as in the amphiphilic core of our particles. However, its fluorescence is effectively quenched when its solubility is decreased as a result of its exposure to water. Thus, a decrease in the fluorescence intensity of Nile red-loaded particles will be a good indication of model drug release. To form the dye-encapsulated particles, the same self-assembly protocol as before was performed except that Nile red was co-dissolved (1% *w*/*w*) with polymer **7** in DMSO. DLS analysis of the resulting sample revealed a slight increase in the size of the particles to 458 nm (Figure 4a). In addition, AFM images of these dye-loaded particles demonstrated that they preserve their spherical morphology upon encapsulation of Nile red (Figure S14). To examine the efficacy of the particles to release their encapsulated cargo in acidic pH under physiologically relevant conditions, they were transferred to a freshly prepared phosphate buffer (pH = 5.5, 10 mM) in a quartz cuvette preheated at 37 °C. Fluorescence spectra were then acquired at specific time points in order to monitor the change in the fluorescence intensity of Nile red as a consequence of any changes to its surrounding environment. As shown in Figure 4b, we observed that about 50% of the dye was released during the first 8 h of incubation at 37 °C, with a maximum of 75% release within 48 h, suggesting the degradation of particles and the subsequent release of the dye.

To investigate the effect of pH in the model drug release, we performed the same experiment at pH = 7.4 (10 mM phosphate buffer) at 37 °C. Surprisingly, a comparable release profile, with slightly slower liberation of Nile red, was observed (Figure 4b). We hypothesise that due to the amphiphilic nature of the core of the particles, water has the ability to penetrate inside and come into close contact with Nile red molecules, altering the surrounding environment and quenching their fluorescence. In addition, such burst release is often observed in polymeric nanoparticulate systems in which the cargo is noncovalently encapsulated in their core [1,2]. In fact, DLS analysis of the dye-loaded particles after 84 h of incubation showed that those in acidic media (pH = 5.5) were completely degraded to their small molecule building blocks (Figure 4a), which is strong evidence of their effective degradation in an acidic aqueous medium, while those at pH = 7.4 kept their integrity, as only minimal change in their size was observed (Figure 4a). Such a small change in the size of particles in buffer at pH = 7.4, compared to DI water, can be attributed to the presence of salts in the buffer solution. This suggests that while the spirocyclic polyacetal-based assemblies are leaky in their nature, the presence of acetal linkages ensures their effective degradation under acidic conditions, which is highly desirable for any drug delivery vehicle to potentially facilitate its clearance from body.

### 3.4. Cytocompatibility and Interactions of Nile Red-Load Particles with Cells

**Figure 5:**
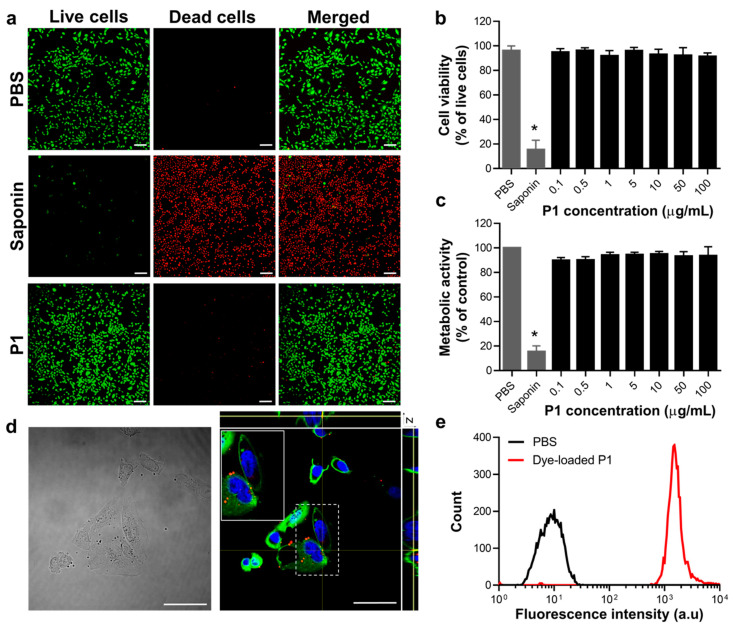
(**a**–**c**) CHO-K1 cells were treated for 24 h with increasing concentrations of **P1** and cell viability was measured by means of the (**a**,**b**) live/dead assay and by (**c**) resazurin-based metabolic activity. (**a**) Representative fluorescence microscopy images showing the distribution of lived (green; calcein AM) and dead (red; ethidium homodimer) cells after treatment with particles (100 μg/mL). The scale bar is 100 µm. (**b**,**c**) The data represent mean ± S.D. of at least three independent experiments performed in triplicate. The results were analyzed using the Student’s t-test and the statistical difference (between vehicle-treated control cells (PBS) and treated cells) was established at * *p* ˂ 0.01. (**d**) Representative differential interference contrast (DIC) and fluorescence confocal microscopy images of CHO-K1 after 4 h incubation with 100 μg/mL of dye-loaded particles (red). The plasma membrane is stained in green and the cell nucleus in blue. The scale bar is 50 μm. (**e**) A flow cytometry diagram showing the plasma membrane adsorption and cellular uptake of dye-loaded **P1**.

Next, we evaluated the cytocompatibility of these particles using CHO-K1, an epithelial cell line commonly used for the evaluation of the toxicity of particles. CHO-K1 cells were incubated with increasing concentrations of **P1**, ranging from 0.1 to 100 μg/mL, for 24 h before measuring cellular viability using the LIVE/DEAD assay and a resazurin-based metabolic activity assay. As observed on fluorescence microscopy images, CHO-K1 cells treated with 100 μg/mL of **P1** showed a similar green/red cell ratio to the vehicle treated cells (PBS) (Figure 5a). The red fluorescence (ethidium homodimer-1) is associated with loss of plasma membrane integrity whereas the green fluorescence (calcein-AM) correlates with intracellular esterase activity of metabolically active cells. In contrast, treatment with 10 μM saponin, used as positive control of cytotoxicity, led to a sharp increase of red-labelled cells and a decrease of green fluorescence. Quantitative analysis of the green/red fluorescence ratio, expressed as % of live cells, indicated no significant decrease in cell viability for all **P1** concentrations evaluated (Figure 5b). Moreover, treatment with **P1** did not reduce the metabolic activity of CHO-K1 cells, as measured by the reduction of resazurin into the highly fluorescent resorufin (Figure 5c). 

Encouraged by these results, using confocal microscopy and flow cytometry, we investigated how the **P1**, loaded with Nile red, interacts with cells and if these particles can be ultimately uptaken by cells. To evaluate plasma membrane adsorption and cell uptake of dye-loaded particles, CHO-K1 cells were incubated with 100 μg/mL of Nile red-loaded assemblies for 4 h at 37 °C. Confocal fluorescence microscopy analysis showed that **P1** gathered at the cell surface, indicating that they bind to the plasma membrane (Figure 5d). Moreover, Z-stack projection also revealed that some red fluorescence puncta of **P1** were located inside the cells, indicating that **P1** were internalized by CHO-K1 cells (Figure 5d). Nonetheless, it is worth mentioning that upon cell uptake likely involving the endosomal-lysosomal pathway, the **P1** undergoes a pH decrease, which could accelerate the release of Nile red and the consequent quenching of its fluorescence. Next, we evaluated the interactions of **P1** with cells by flow cytometry and we observed that, despite extensive washes of cells before analysis, the majority of CHO-K1 cells were highly fluorescent (Figure 5e). Overall, these studies revealed that spirocyclic polyacetal-based assemblies are fully cytocompatible, interact avidly with the plasma membrane and can be ultimately uptaken by cells.

## 4. Conclusions

In summary, we demonstrated a Cu-catalyzed alkyne–azide “click” polymerization approach to synthesize amphiphilic spirocyclic polyacetals using diazide-functionalized PEG and dialkyne-decorated spirocyclic acetal monomers. The resulting polymers exhibit low T_g_ values rendering them amorphous at ambient temperature. Degradation of these polymers in acidic CDCl_3_ showed a gradual decomposition of the starting polymers and formation of their starting constituents as evidenced by ^1^H NMR. In addition, it was shown that these amphiphilic spirocyclic polyacetals are able to undergo self-assembly in DMSO/H_2_O mixture to form particles with diameters of ~400 nm. Furthermore, by means of Nile red encapsulation and in vitro release experiments, we demonstrated that these materials possess an amphiphilic core, rendering them leaky at neutral pH with the preservation of particle integrity, as evidenced by DLS measurements. Nevertheless, efficient Nile red release and the complete degradation of these materials were achieved at pH = 5.5. To show the potential application of this system in nanomedicine, cellular assays demonstrated the nontoxic nature of these self-assembled materials. Moreover, these particles bind to the cell surface and can be taken up by CHO-K1 cells and ultimately transport their encapsulated cargo into cells. Our results show that these spirocyclic polyacetal-based particles are promising candidates for biomedical applications.

## Data Availability

Data presented in this study are available upon request from the corresponding author.

## Supplementary Materials

The following are available online at https://www.mdpi.com/2079-4991/11/1/161/s1, Figure S1–S10: ^1^H- and ^13^C-NMR spectra for compounds **2**–**6** and polymer **8**; Figure S11: pH-triggered degradation of polymer **8** in 80 mM trifluoroacetic acid in CDCl_3_ monitored by ^1^H NMR spectroscopy; Figure S12: Stability of **7** in non-acidic CDCl_3_ monitored by ^1^H NMR spectroscopy; Figure S13: Refractive index (RI) traces in the SEC analysis of (a) polymer **7** before and after degradation as well as the PEG starting material (*M*_n_ = 400 g/mol) and (b) polymer **8** before and after degradation as well as the PEG starting material (*M*_n_ = 600 g/mol); and Figure S14: AFM image of Nile red-loaded particles (P1) formed by polymer **7**.
