# Supplementary material for: Degradable Spirocyclic Polyacetal-Based Core-Amphiphilic Assemblies for Encapsulation and Release of Hydrophobic Cargo
Source: Nanomaterials (Basel). 2021 Jan 10;11(1):161. doi: 10.3390/nano11010161 (PMC7826923; doi:10.3390/nano11010161)
Supplement: Supplementary file 1 [file nanomaterials-11-00161-s001.pdf]

nanomaterials

MDPI

Supplementary Materials

Degradable Spirocyclic Polyacetal-Based Core-Amphiphilic Assemblies for Encapsulation and Release of Hydrophobic Cargo

Brandon Andrade-Gagnon, Marilyne Bélanger-Bouliga, Phuong Trang Nguyen, Thi Hong Diep Nguyen, Steve Bourgault and Ali Nazemi *

Department of Chemistry, Université du Québec à Montréal, C.P.8888, Succursale Centre-Ville, Montréal, QC H3C 3P8, Canada; andrade-gagnon.brandon@courrier.uqam.ca (B.A.-G.); belanger-bouliga.marilyne@courrier.uqam.ca (M.B.-B.); phuong.t.ngn@gmail.com (P.T.N.); nguyen.thi_hong_diep@courrier.uqam.ca (T.H.D.N.); bourgault.steve@uqam.ca (S.B.)

* Correspondence: nazemi.ali@uqam.ca

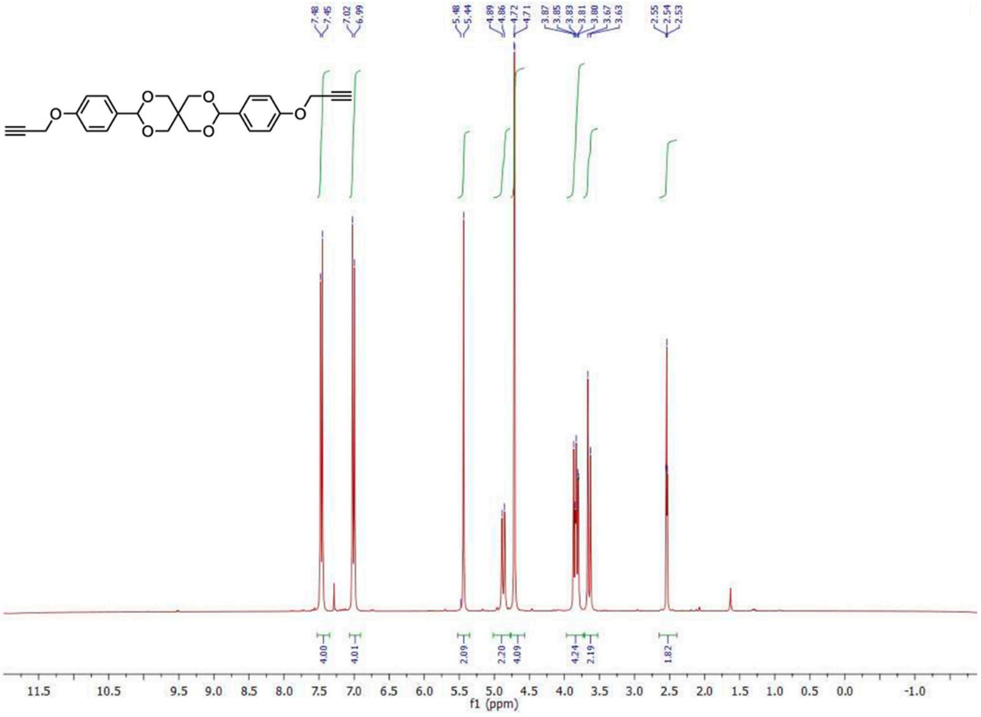

Figure S1. ^{1}H NMR spectrum of compound **2** (300 MHz, $CDCl_3$).

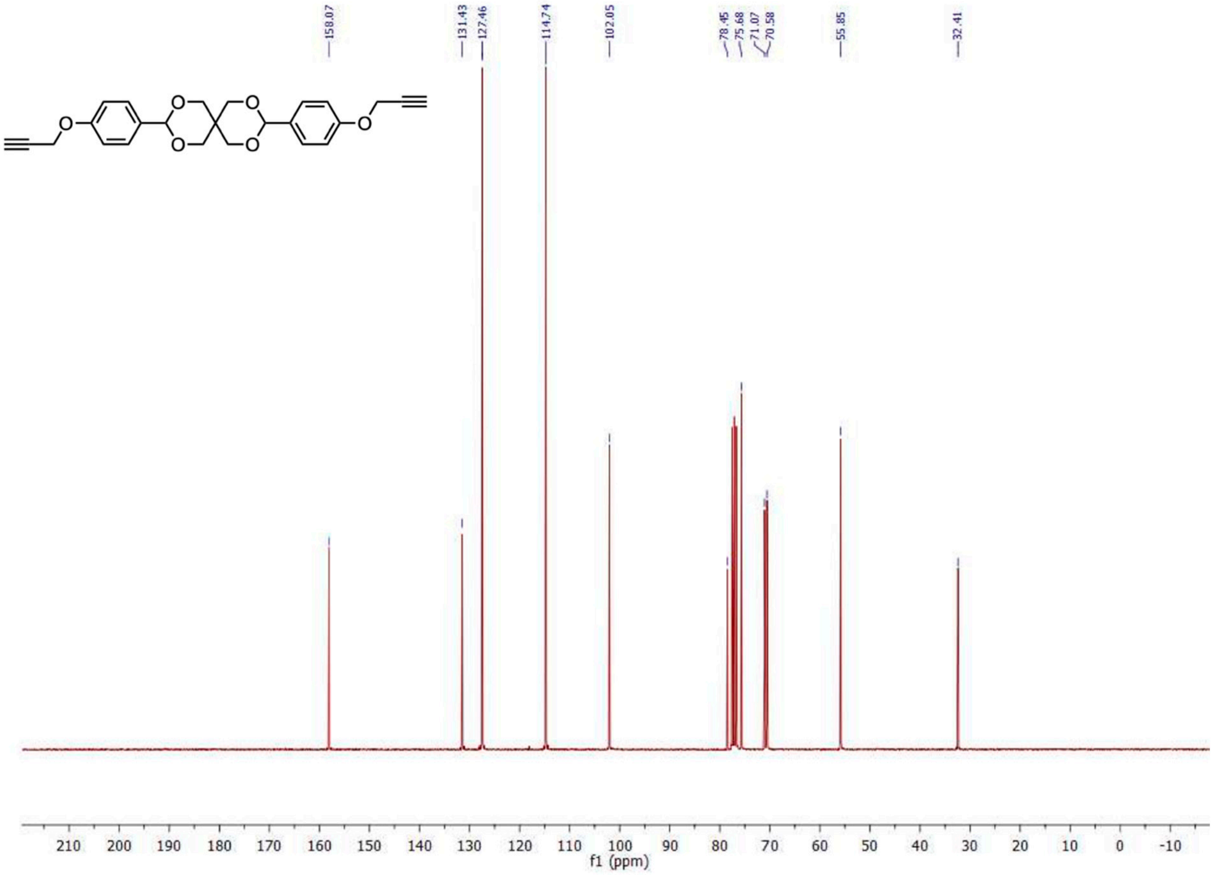

Figure S2. ^{13}C NMR spectrum of compound **2** (75 MHz, $CDCl_3$).

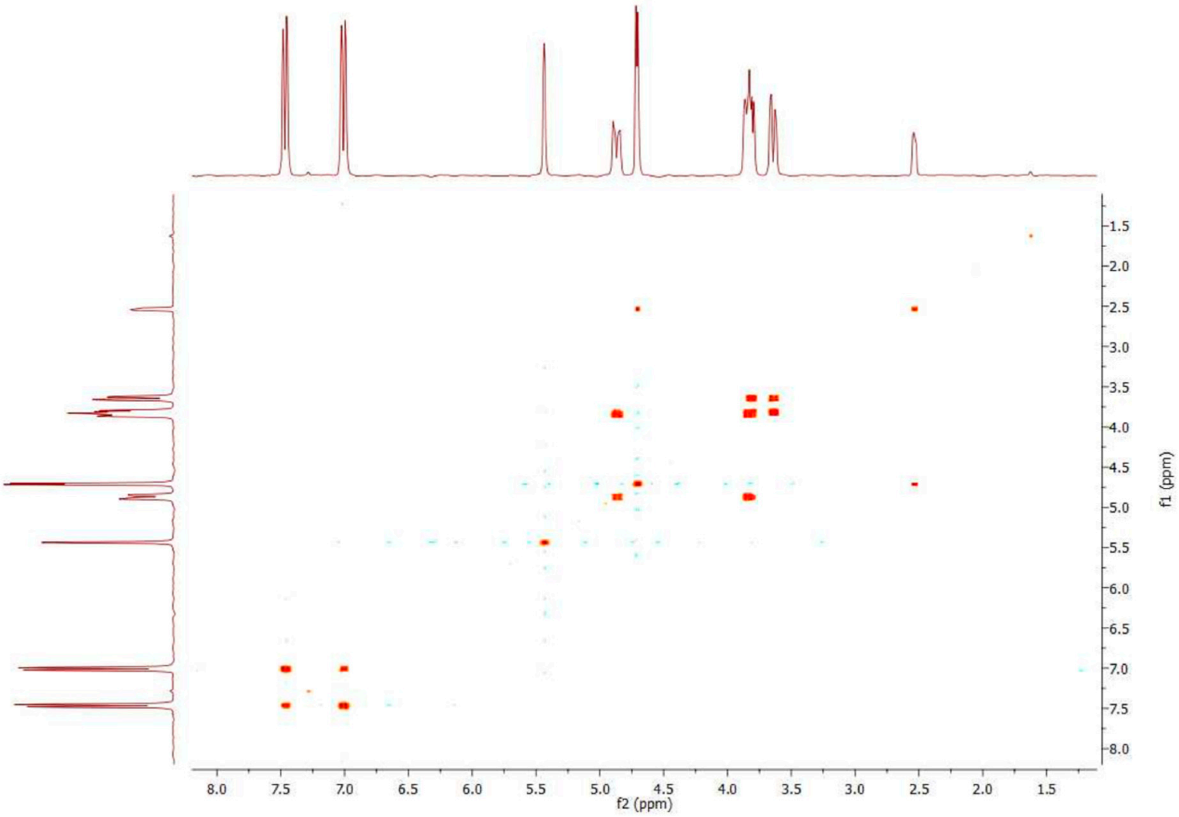

Figure S3. 2D ^{1}H– ^{1}H COSY spectrum of compound **2** (300 MHz, $CDCl_3$).

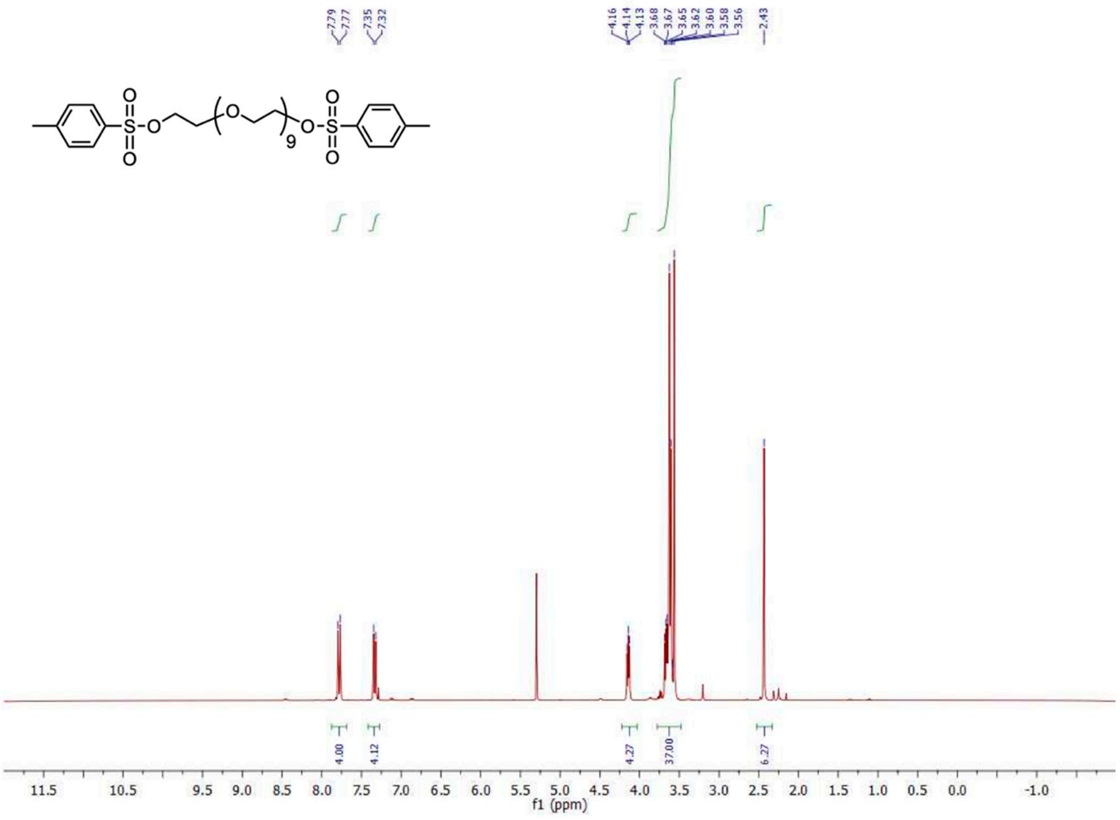

Figure S4. ^{1}H NMR spectrum of compound **3** (300 MHz, $CDCl_3$).

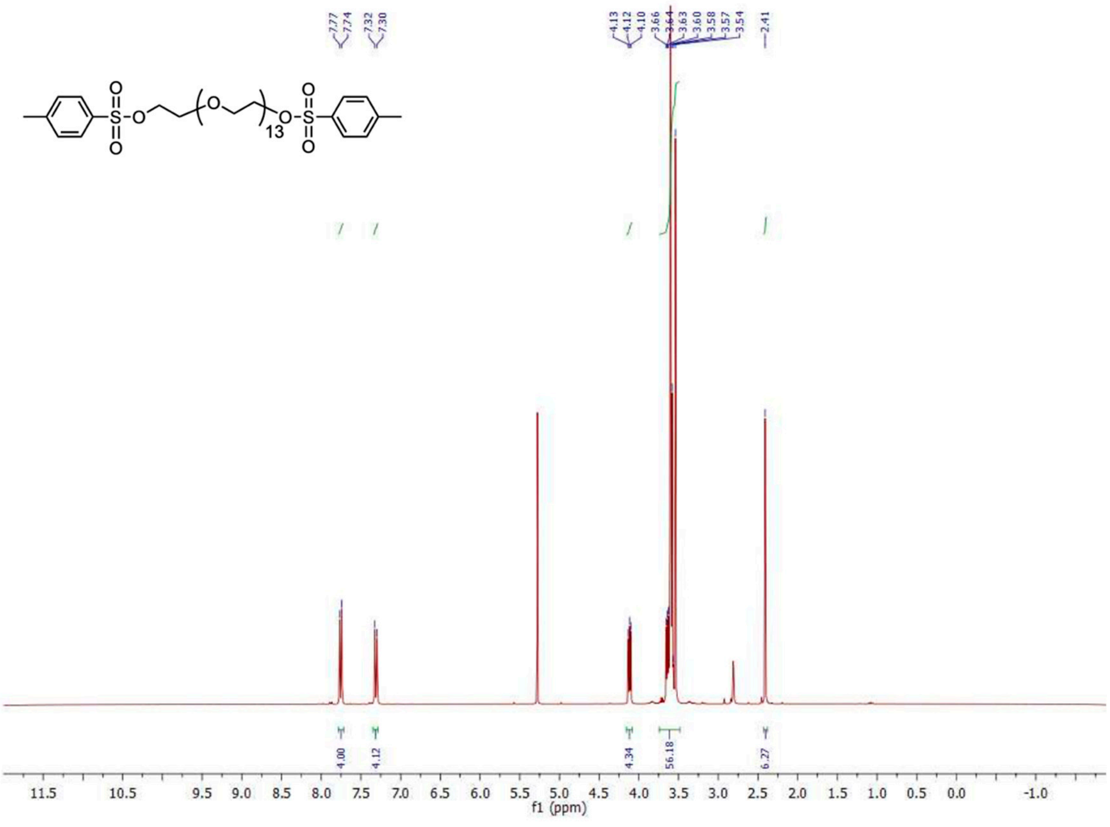

Figure S5. ^{1}H NMR spectrum of compound **4** (300 MHz, $CDCl_3$).

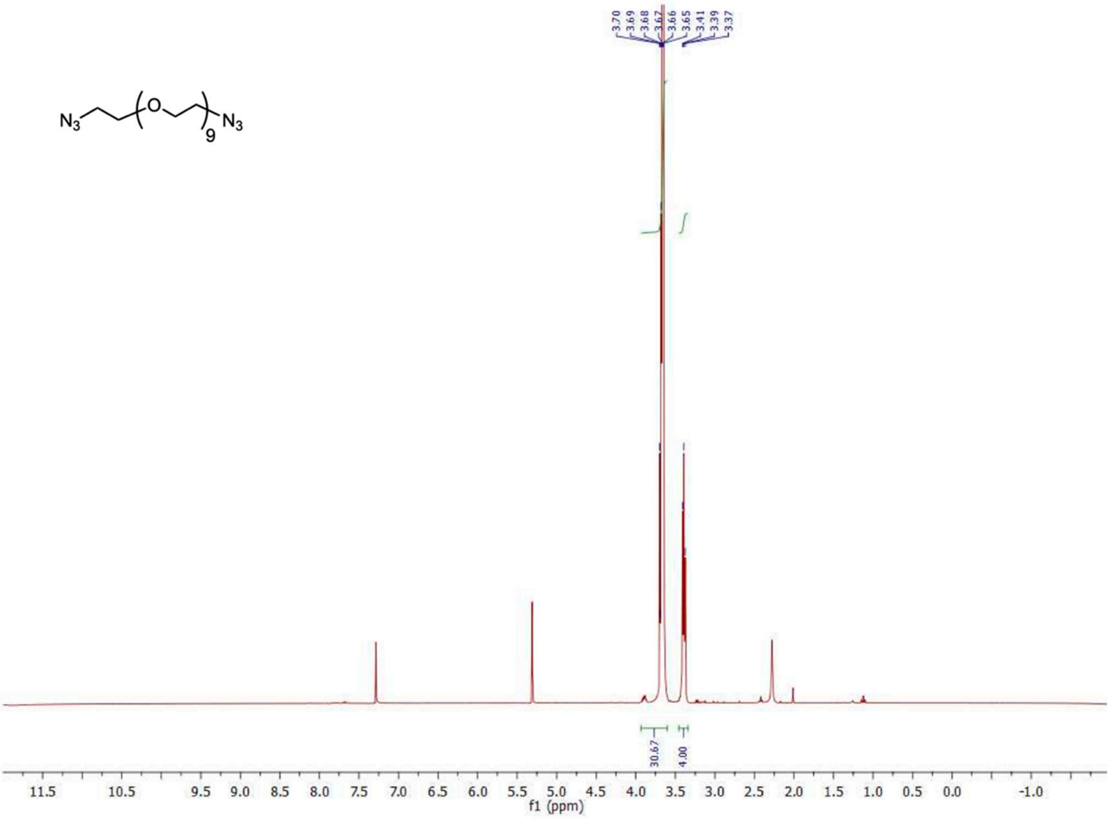

Figure S6. ^{1}H NMR spectrum of compound **5** (300 MHz, $CDCl_3$).

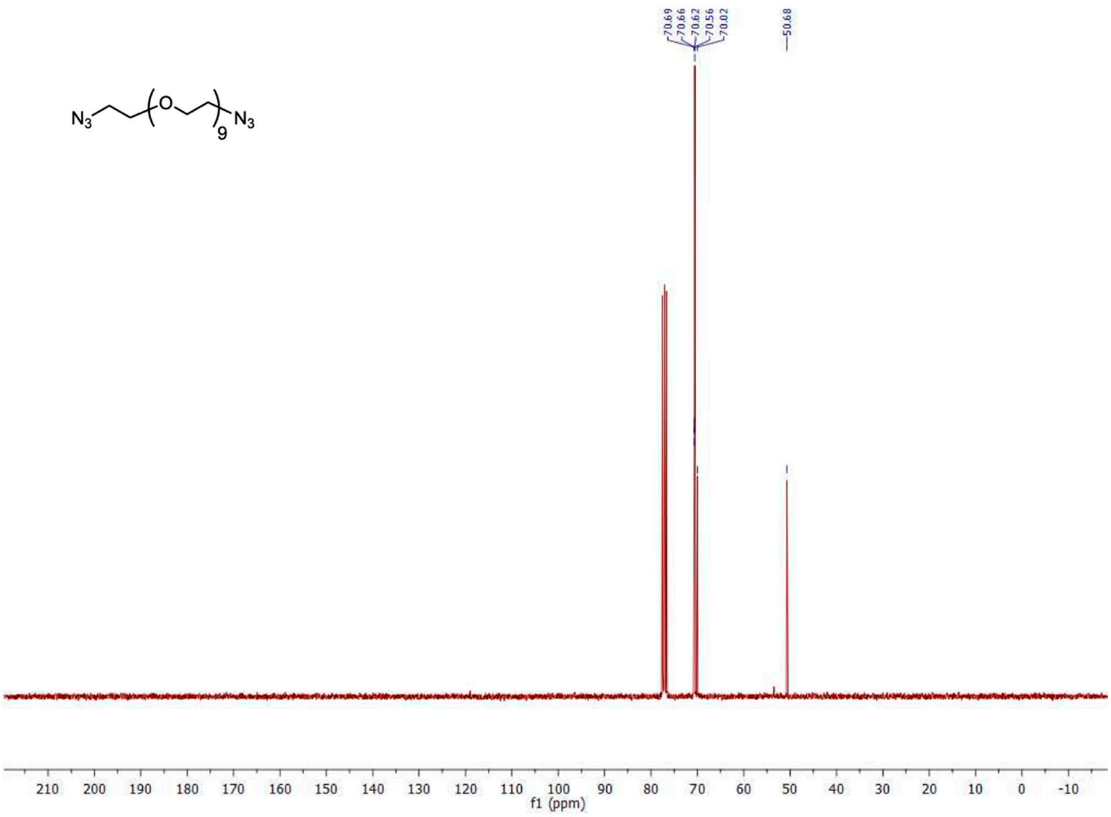

Figure S7. ^{13}C NMR spectrum of compound **5** (75 MHz, $CDCl_3$).

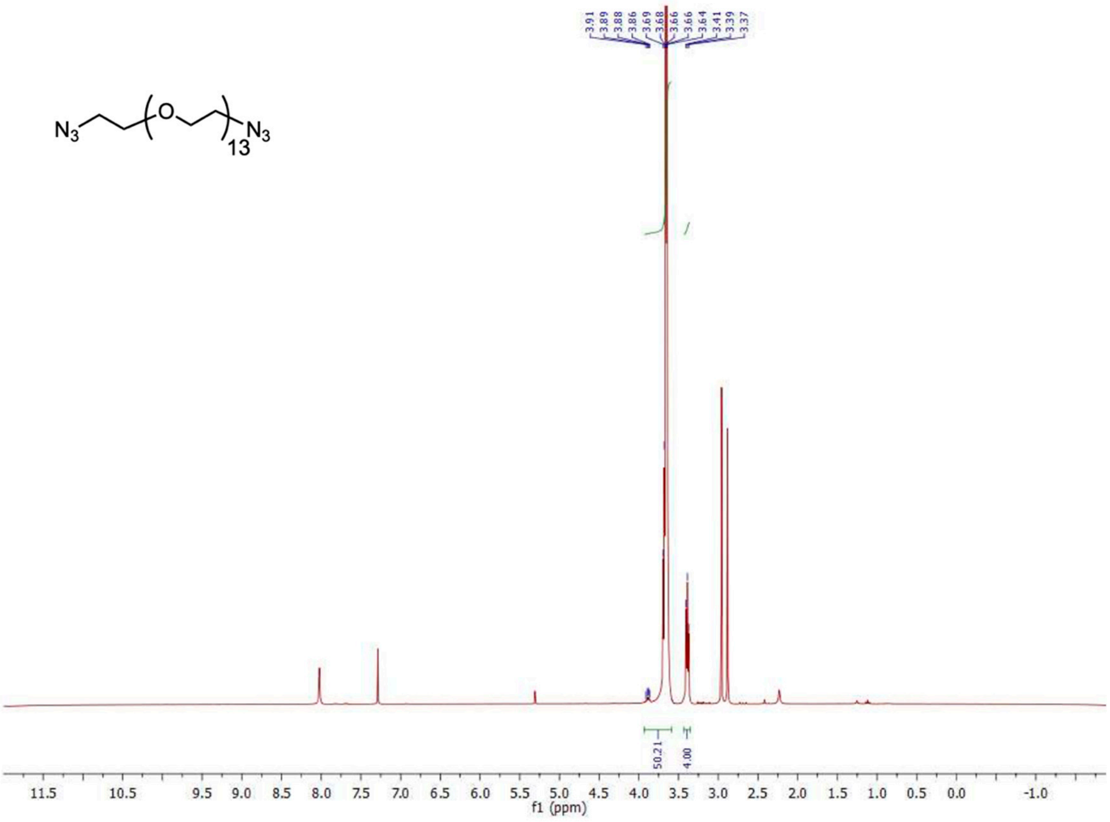

Figure S8. ^{1}H NMR spectrum of compound **6** (300 MHz, $CDCl_3$).

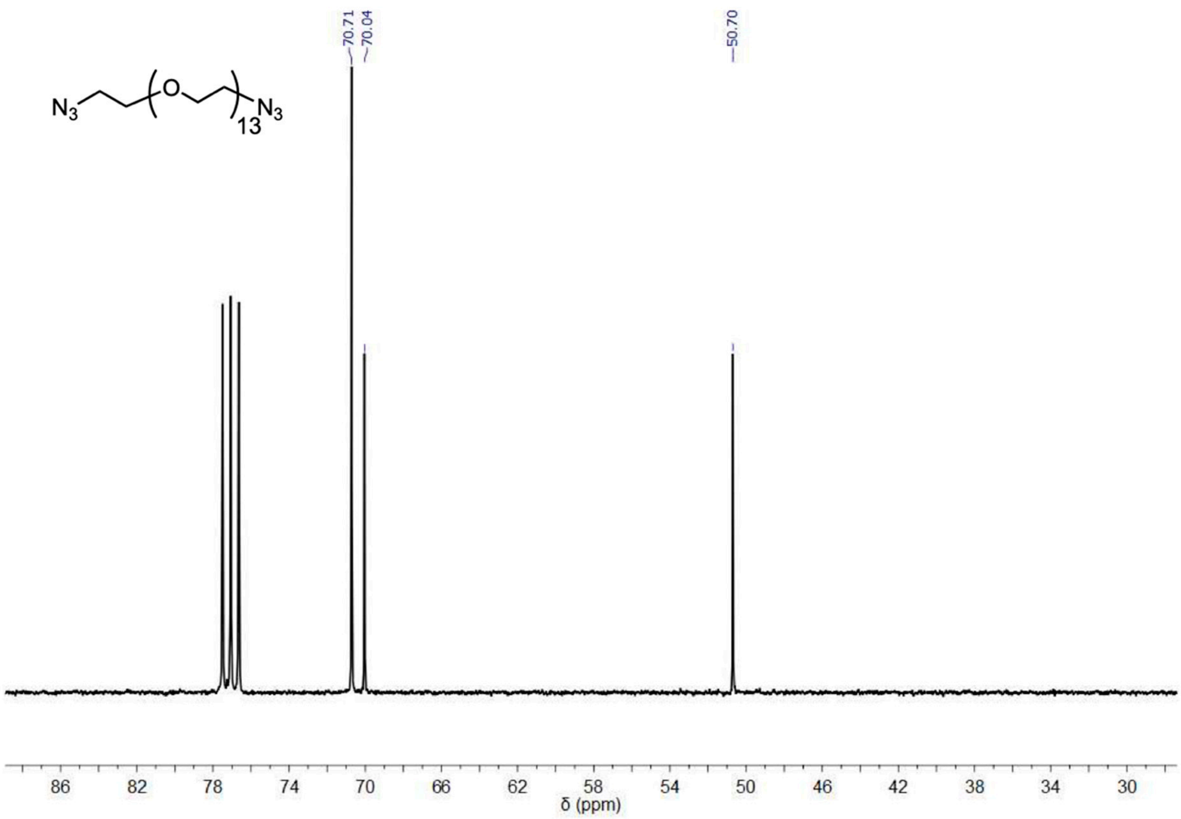

Figure S9. ^{13}C NMR spectrum of compound **6** (75 MHz, $CDCl_3$).

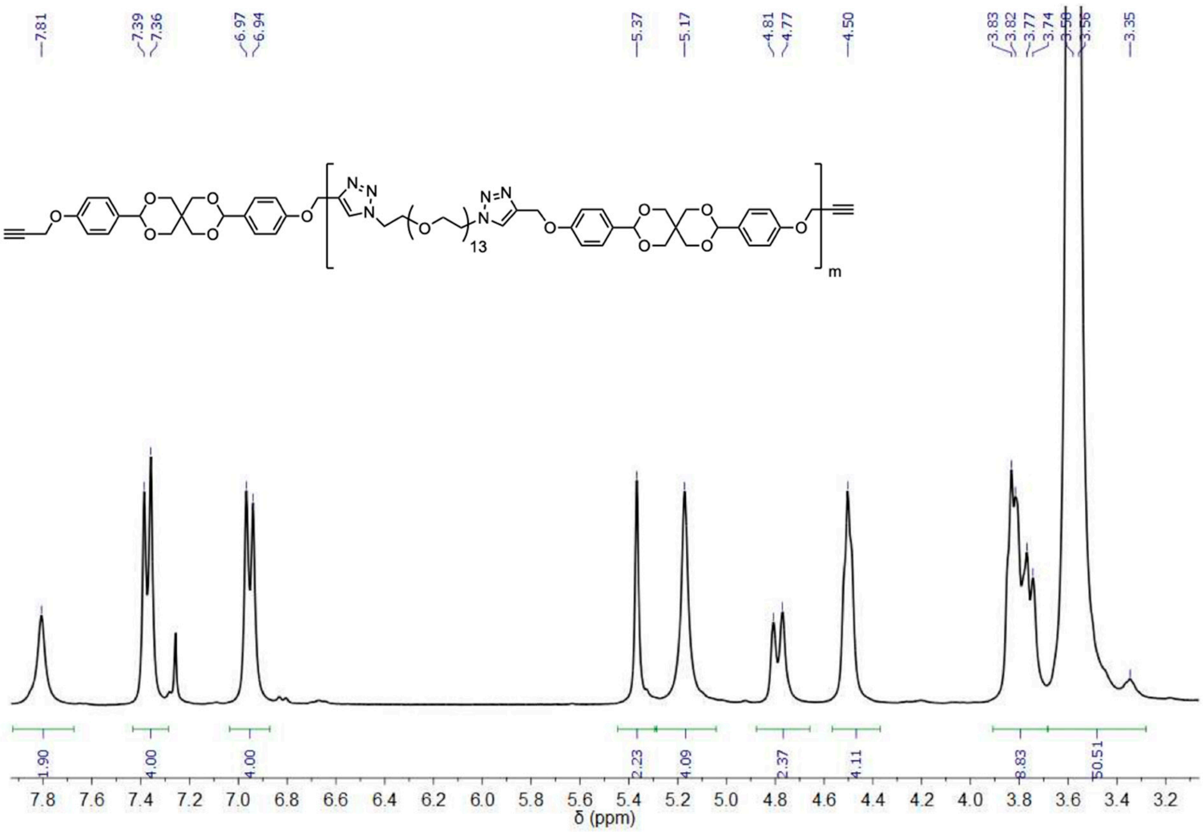

Figure S10. ^1H NMR spectrum of polymer **8** (300 MHz, $CDCl_3$).

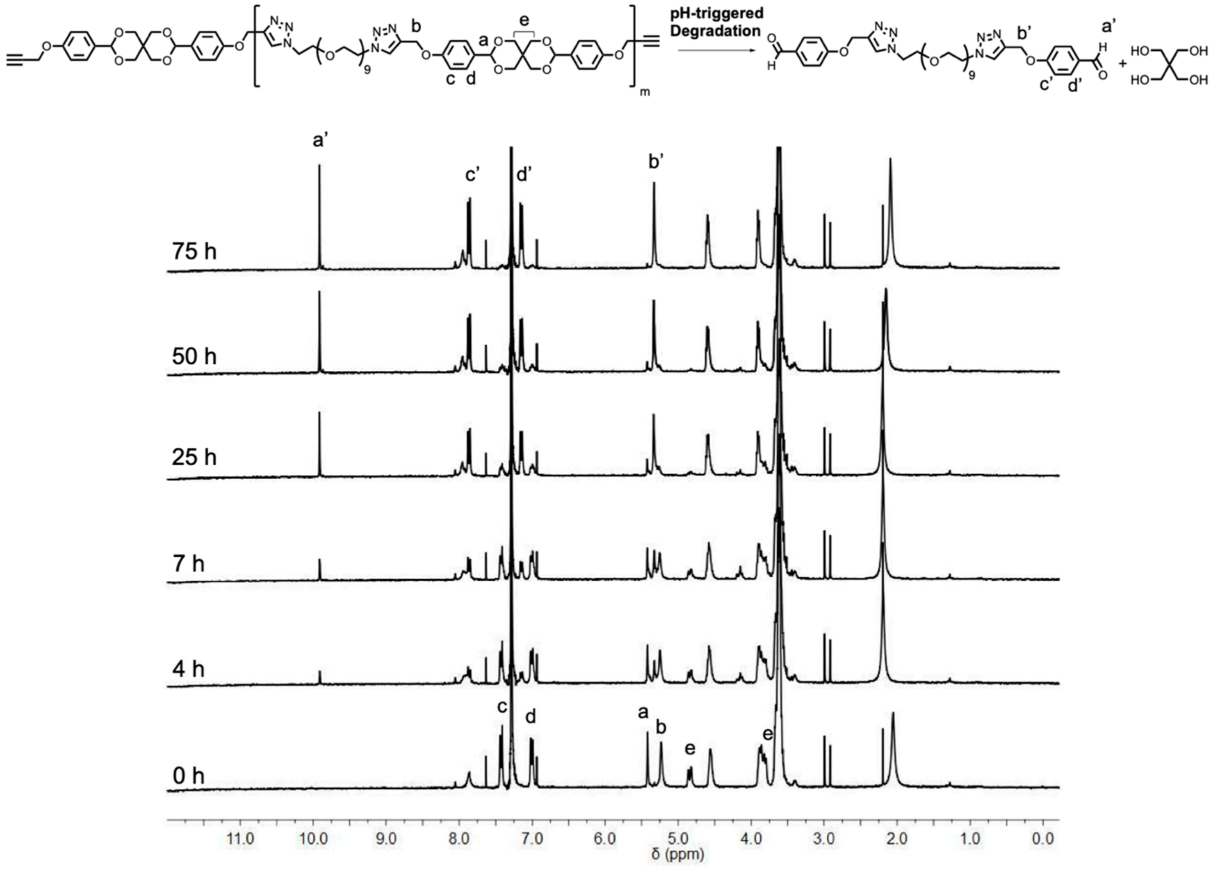

Figure S11. pH-triggered degradation of polymer **8** in 80 mM trifluoroacetic acid in $CDCl_3$ monitored by ^1H NMR spectroscopy.

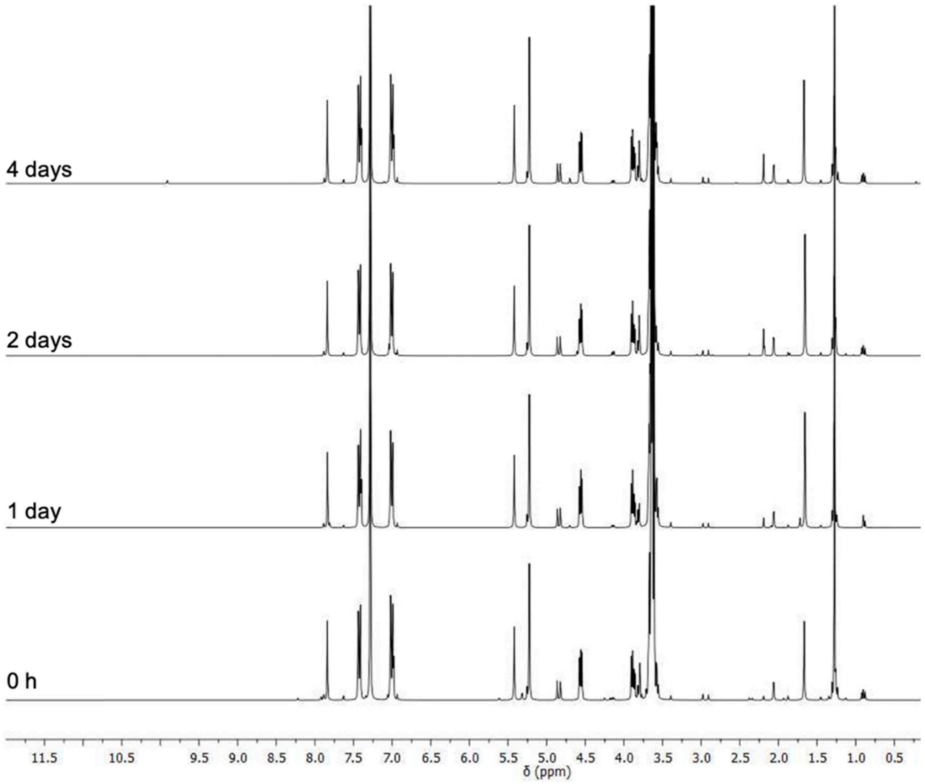

Figure S12. Stability of **7** in non-acidic $CDCl_3$ monitored by ^1H NMR spectroscopy.

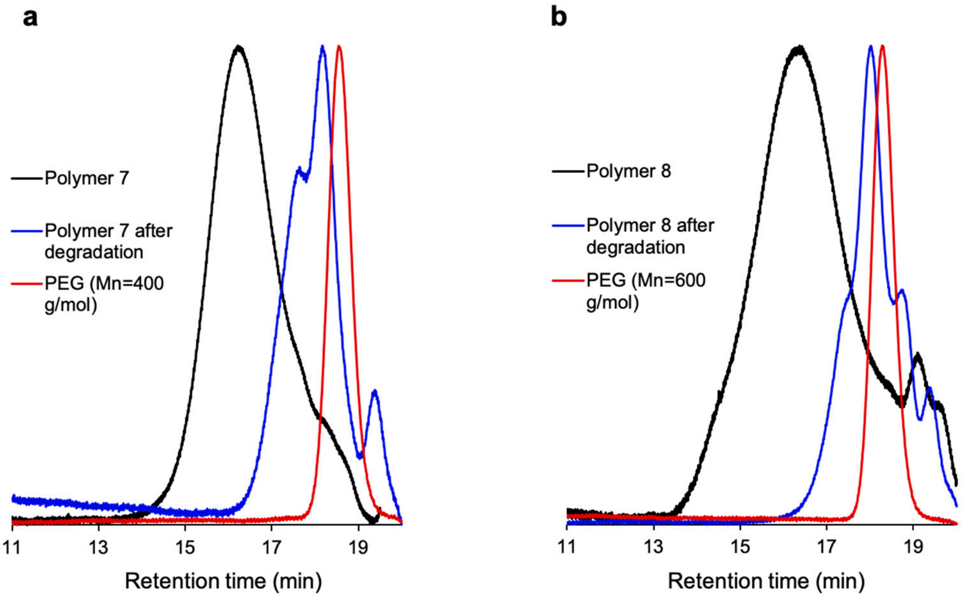

Figure S13. Refractive index (RI) traces in the SEC analysis of (**a**) polymer **7** before and after degradation as well as the PEG starting material (M_n = 400 g/mol) and (**b**) polymer **8** before and after degradation as well as the PEG starting material (M_n = 600 g/mol).

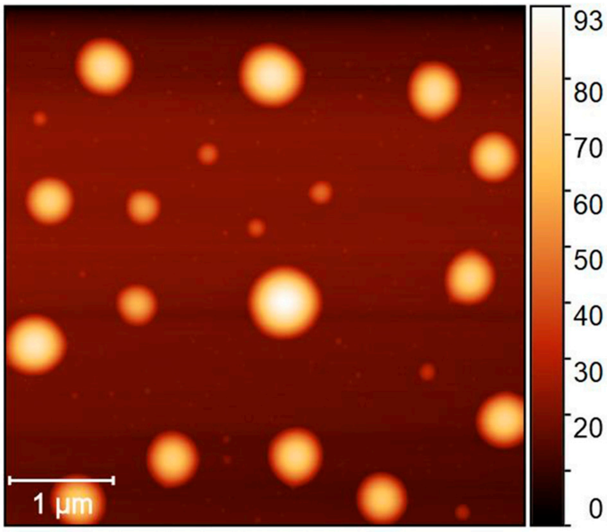

Figure S14. AFM image of nile red-loaded particles (**P1**) formed by polymer **7**.

© 2021 by the authors. Licensee MDPI, Basel, Switzerland. This article is an open access article distributed under the terms and conditions of the Creative Commons Attribution (CC BY) license (http://creativecommons.org/licenses/by/4.0/).
